# Amelioration of Hepatotoxic and Neurotoxic Effect of Cartap by *Aloe vera* in Wistar Rats

**DOI:** 10.3390/toxics11050472

**Published:** 2023-05-22

**Authors:** Vivek Kumar Gupta, Uichang Park, Nikhat J. Siddiqi, Yun Suk Huh, Bechan Sharma

**Affiliations:** 1 Department of Biochemistry, University of Allahabad, Prayagraj 211002, India; vivekgupta@inha.ac.kr; 2 NanoBio High-Tech Materials Research Center, Department of Biological Sciences and Bioengineering, Inha University, Incheon 22212, Republic of Korea; 3 FCSM-Department of Biochemistry, King Saud University, Riyadh 11495, Saudi Arabia

**Keywords:** cartap, hepatotoxicity, neurotoxicity, antioxidants, histopathology, *Aloe vera*

## Abstract

Pesticide exposure can pose a serious risk to nontarget animals. Cartap is being broadly used in agricultural fields. The toxic effects of cartap on the levels of hepatotoxicity and neurotoxicity have not been properly studied in mammalian systems. Therefore, the present work focused on the effect of cartap on the liver and brain of Wistar rats and made an assessment of the ameliorating potential of *A. vera*. The experimental animals were divided into 4 groups, comprising six rats in each: Group 1—Control; Group 2—*A. vera*; Group 3—Cartap; and Group 4—*A. vera* + Cartap. The animals orally given cartap and *A. vera* were sacrificed after 24 h of the final treatment and histological and biochemical investigations were conducted in liver and brain of Wistar rats. Cartap at sublethal concentrations caused substantial decreases in CAT, SOD, and GST levels in the experimental rats. The activity levels of transaminases and phosphatases in cartap group were also found to be substantially altered. The AChE activity was recorded as decreasing in RBC membrane and brain of the cartap-treated animals. The TNF-α and IL-6 level in serum were increased expressively in the cartap challenged groups. Histological investigation of liver showed disorganized hepatic cords and severely congested central veins due to cartap. However, the *A. vera* extract was observed to significantly protect against the effects of cartap toxicity. The protective impact of *A. vera* against cartap toxicity may be due to the existence of antioxidants in it. These findings suggest that *A. vera* may be developed as a potential supplement to the appropriate medication in the treatment of cartap toxicity.

## 1. Introduction

Carbamates are chemical compounds that are commonly used for monitoring insects and pests in farming practices [1]. The extent of carbamates has been detected in environmental components, and also in food as a main contaminant [1,2]. According to an estimate, a far smaller amount of the total pesticides sprayed is effectively utilized for killing the insects and pests, whereas the majority of it reaches the environment [3]. It has been described that carbamate exposure induces numerous adverse effects by inducing oxidative stress in the exposed animals by modulating the functions of various cellular constituents in their key organs, such as the melatonin receptors [4] and the nervous system, via alterations in acetylcholinesterase (AChE) activity [5]. In comparison to the other pesticides, carbamates have less toxicity to the non-target animals [6]. Cartap is a thiocarbamate insecticide that primarily works against the brain function [7,8]. Cartap is extensively used in farming fields to control pests and to increase the crop’s yield. However, its frequent and non-judicious application may result in the adulteration of non-target animals, including humans. Although cartap toxicity has been considered to be marginal in comparison to other carbamates, it has been reported to exert high mammalian toxicity [9,10]. Its primary action is mediated by the inhibition of the acetylcholine receptor [11]. Cartap can disrupt the Na^+^/K^+^ balance in neurons, forcing them to conduct signals constantly, which leads to the development of neurodegenerative disorders. Cartap can reversibly bind with the active site of AChE [12]. Further, cartap has been shown to induce apoptosis in C2C12 cells by excess production of ROS [11]. Although the antioxidative systems of cells are able to quench and scavenge the ROS and lower their damaging capabilities, excess production of free radicals can generate a difference in the oxidants/antioxidants ratio. This can result in the development of oxidative stress and cause damage to the different biomolecules [13,14]. The toxic impact of cartap was also reported on several non-target aquatic organisms such as *Anabaena variabilis* [15], *Labeo rohita* [16], *Daphnia magna* and *Oryzias latipes* [8]. There are inadequate reports available on the effect of cartap on the levels of toxic impacts in rats. Therefore, the present study was designed to assess the effects of cartap in key rat organs.

Herbal remedies have been an important part of Indian ancient medicinal systems since the development of primitive civilizations. Herbal medicines are cheaper in cost and have the fewest side effects. Herbs are well-defined as the plants that are used for their aromatic properties [17] and serve as a great source of phytochemicals with impressive antioxidant potential [18]. Plant-based medicines are the richest sources of biologically active metabolites including antioxidants, and they can potentially be used as a viable supplement to the numerous neurotoxicity and liver disorders [19,20]. Plant-based products including *Aloe vera (A. vera)* are a valuable source of antioxidants [21]. *A. vera* has been reported to contain numerous secondary metabolites and antioxidant agents [22,23]. The antioxidants present in *A. vera* constitute chemical ingredients with immense free radical-scavenging abilities. In addition, the plant exhibits several pharmacological properties that act as antimicrobial, immune booster, antitumor, hypoglycemic, hypolipidemic, wound healing, antiseptic, cosmetic, and anti-inflammatory agents [24]. Thus, *A. vera* formulations can act as multifaceted therapeutic components active against various disorders.

The substantial literature survey suggested that the carbamates exhibited the capacity to cause stress in living systems. However, the protective role of *A. vera* against cartap-induced toxicity in mammalian systems has not been investigated. The current study was thus commenced to delineate the effects of cartap and *A. vera* in rats.

## 2. Materials and Methods

### 2.1. Chemicals

Cartap (S.C.C. LTD) was purchased from Dhanuka Agritech Limited, Haryana, India. ATI and H_2_O_2_ were obtained from TCI Chemicals (India) Pvt. Ltd. Follin’s reagent, NADH, GSH, DTNB, NaCl, and BSA were bought from SRL Pvt. Ltd., Mumbai, India. Triton X-100, pyrogallol, EDTA, CH_3_COONa, Na_2_CO_3_, NaHCO_3_, Na_2_HPO_4_, NaH_2_PO_4_, and NaOH were purchased from MERK.

### 2.2. Collection of A. vera Leaf and Phytochemical Extraction

Young and fresh leaves from the *A. vera* plant were acquired from the garden of our university and freed from any other particles. The leaves were collected in January 2022. The young leaves were dried up in shadow at 25 °C and pulverized, and then the phytochemicals were extracted (1:10 *w*/*v*) in H_2_O using a Soxhlet apparatus, freeze-dried and kept at −20 °C.

### 2.3. Animal Care and Experimental Design

The 8-week-aged male Wistar rats, weighing about 150 ± 10 g, were acquired from CDRI-Lucknow, UP, India. All rats were acclimatized to the environment in polypropylene cages in standard conditions. Each acclimatized group contained 6 rats. All the rats had free access to water and food. The animals were treated with 10% LD_50_ of cartap, followed by oral gavage of *A. vera* extract. The rats were divided into 4 groups. Group 1—control; Group 2—*A. vera* treated (420 mg kg^−1^ bw); Group 3—cartap (29 mg kg^−1^ bw); and Group 4—*A. vera* extract (420 mg kg^−1^ bw) followed by cartap (29 mg kg^−1^ bw). The oral administration of cartap and *A. vera* extract was carried out for the experimental animals until 15 days after every 24 h of gap. The rats from all groups were euthanized and sacrificed; this was followed by the collection of blood, liver, and brain tissues, and these were stored at −20 °C. The study was designed and executed as per the IAEC guidelines of the University of Allahabad (CPCSEA: 839/GO/Re/04/CPCSEA).

### 2.4. Preparation of Post-Nuclear Supernatant (PNS) in Sucrose Solution

The liver and brain tissues were homogenized (10% *w*/*v*) in a 0.25 M sucrose buffer and centrifugated at 9000× *g* for 30 min at 4 °C. The PNSs were stored for biochemical estimations.

### 2.5. AChE Extraction from RBC and Rat Brain

The extraction of AChE enzyme from erythrocyte membrane and brain tissue was performed via the procedures described earlier by Gupta et al. in 2022 [8]. The homogenates were prepared in a phosphate buffer (50 mM; pH 7.4) containing Triton X-100 (0.2%) followed by centrifugation at 10,000× *g* at 4 °C for 10 min. The resulting supernatants containing AChE were used for the enzyme assay.

### 2.6. Estimation of Malondialdehyde (MDA) and Glutathione (GSH) Level

The levels of MDA and GSH were estimated in the supernatants of rat liver, and brain [25], respectively. The absorbance of the colored reaction mixture was monitored using a UV-visible spectrophotometer and the units of results were shown in terms of nmol MDA/mg protein for MDA and µg GSH/mg protein for reduced GSH.

### 2.7. Activity Assay for Superoxide Dismutase (SOD), Catalase (CAT), and Glutathione-S-Transferase (GST)

The specific activity of SOD, CAT and GST was assessed by following the procedures of Marklund and Marklund (1974) [26], Beers and Sizer (1952) [27] and Habig et al. (1974) [28], respectively, in the PNS of the liver and brain. The absorbance of the reaction mixture was recorded using a UV-visible spectrophotometer, and the unit for the activities of enzymes were presented as IUmg^−1^ protein.

### 2.8. Estimation of TNF-α and IL-6

The levels of TNF-α and IL-6 were estimated in the serum of rats exposed to cartap and *A. vera* by following the procedure described in the kit used (Krishgen Biosystems, Mumbai, India).

### 2.9. Calculation for Oxidative Stress Index

The extent of oxidative stress was calculated as the correlation of prooxidant/antioxidant (P/A) in liver and brain tissues. This was performed using following formulae:

P/A = Levels of MDA/Activities of SOD + CAT + GST

### 2.10. Assay for Transaminases (SGOT and SGPT) and Phosphatases (ALP and ACP)

The activities of phosphatases [29] and transaminases [30] were determined in the blood serum. The change in absorbance of colored reaction mixture was monitored using a UV-visible spectrophotometer. The units of specific activities of enzymes were presented as IUmg^−1^ protein.

### 2.11. Assay for Acetylcholinesterase (AChE) Activity

The specific activities of AChE were evaluated using the PNS of RBC and rat brain [31]. The specific activity was computed, and the results were presented as µmoles ATI hydrolyzed ml^−1^ min^−1^ or as IUmg^−1^ protein.

### 2.12. Estimation of Total Protein Contents (TPCs)

The TPCs in the PNS of liver, brain and blood sera were determined via the method of Lowry et al. (1952) [32].

### 2.13. Histological Study of Liver

The histological investigation of liver tissue was performed via the procedure explained by Gupta et al. in 2019 [33]. In brief, the liver tissues were quickly removed; this was followed by washing with PBS and blotting. The tissue fixation was performed in Bouin’s solution; the tissue was washed under running water; this was followed by the dehydration of tissues and their embedding in a wax block. The tissues embedded in wax were sectioned (10 μm) serially by the rotary spencer microtome and stretched on the albumin-smeared sterilized glass slides, followed by staining. The eosin-hematoxylin staining method was performed to stain the tissue section. The slides holding tissue sections were inspected under light microscope after mounting with dibutylphthalate polystyrene xylene.

### 2.14. Statistical Analysis

Graph Pad Prism version 5.01 was used for data analysis using ANOVA (one-way). The histological slides of liver sections were analyzed employing the Dewinter digital camera-assisted microscope (model-YJ-2016). The spectrophotometric assays were conducted using the ThermoScientific Spectroscan UV2700 UV-visible spectrophotometer. The results were found to be significant at *p* ≤ 0.05.

## 3. Results

### 3.1. Effect of Cartap and A. vera on the Levels of MDA and GSH

**Figure 1:**
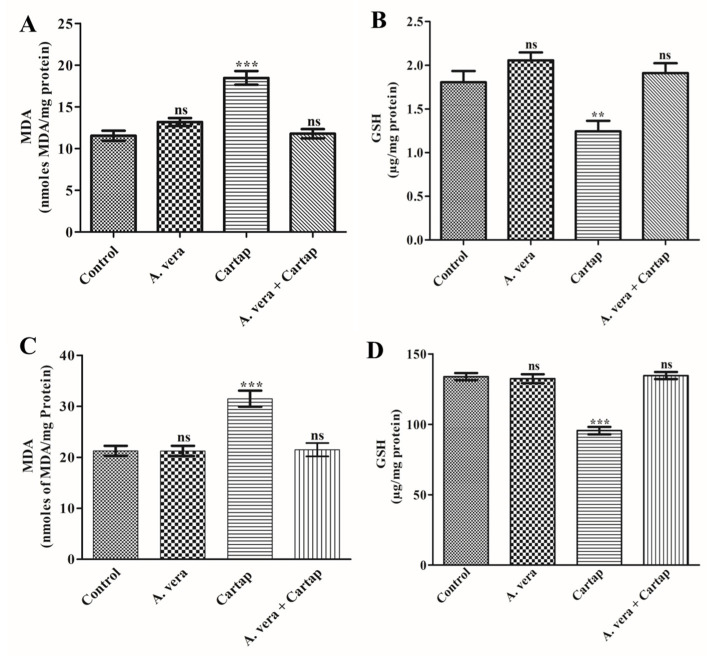
Influence of cartap and *A. vera* on the contents of (**A**) MDA in brain, (**B**) GSH in brain, (**C**) MDA in liver and (**D**) GSH in liver tissues. The treatment procedures were performed as described in Section 2. The unit for the level of MDA was denoted as nmoles MDAmg^−1^ protein. The level of GSH was expressed as µg/mg protein. The values presented were mean ± SD of 3 investigations and significant at *p* ≤ 0.05 in comparison to the control. The (+) and (−) signs show a rise or decline in the contents of the indices. MDA: malondialdehyde; GSH: glutathione; **, ***: Level of significance; ns: non-significant.

In an effort to evaluate the impact of cartap, the treatment of rats was performed as mentioned in Section 2. The oral treatment of rats with the *A. vera* was also performed in order to evaluate its protective effect. The changes in the levels of MDA and GSH in the liver and brain of cartap-treated rats (Figure 1). Cartap caused a significant rise in the contents of MDA by 48.11% and decrease in the GSH by 28.64% in liver tissues. The exposure of rats to cartap also enhanced the content of MDA by 60.10% and GSH by 31.17% in brain tissues. These indices did not significantly change in liver (MDA by 0.094% and GSH by 1.12%) or brain (MDA by 4.42% and GSH by 2.43%) tissues after treatment with *A. vera* alone. The MDA and GSH levels in the liver and brain tissues of cartap-treated rats were found to be relatively equal to those of the control after pre-treatment with the *A. vera* extract.

### 3.2. Impact of Cartap and A. vera on the Activities of SOD, CAT, and GST

**Figure 2:**
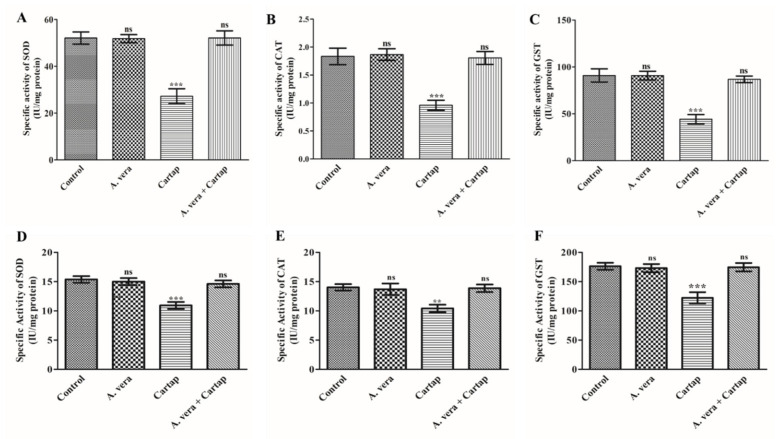
Impact of cartap and *A. vera* on the activity of (**A**) SOD; (**B**) CAT; and (**C**) GST in liver and (**D**) SOD; (**E**) CAT; and (**F**) GST in rat brain. The methods for cartap and *A. vera* extract treatment and the activity assays of enzymes were as stated in Section 2. The units of enzymes activities were termed as IUmg^−1^ protein. Abbreviations: **, ***: Level of significance; ns: non-significant.

To determine the effect of cartap on the SOD, CAT and GST activities, their activity assays were conducted using the PNS of liver and brain tissues from rats treated for 15 days, as explained in Section 2. The results showed that cartap caused a significant decline in the levels of SOD, CAT, and GST in liver and brain tissues (Figure 2). The percentage decrease in the activities of SOD, CAT, and GST was calculated to be 47.72, 47.64 and 51.30% in liver and 28.93, 25.80, and 30.59% in brain, respectively, in cartap-treated group. The pre-administration of *A. vera* to the cartap-exposed rats showed protection from pesticide’s toxic effects. The activities of enzymes were entirely protected by plant extract (Figure 2) treatment in both liver and brain tissue. The pretreatment of *A. vera* itself did not show any significant alterations in the activities of enzymes.

### 3.3. Influence on the Level of P/A Ratio

The ratio of P/A has been referred to as the oxidative stress index (OSI). The levels of OSI were computed in cartap-treated rats for 15 days as described in Materials and Methods. The P/A ratio was found to be greater in the liver (197.26%) as compared to the brain (129.46%) of rats treated with cartap (Table 1). The data suggested that the *A. vera* was able to potentially ameliorate the oxidative stress as the P/A values were found to be almost equal to the control values.

### 3.4. Effects of Cartap and A. vera on the Concentration of Proinflamatory Cytokines (TNF-α and IL-6)

**Figure 3:**
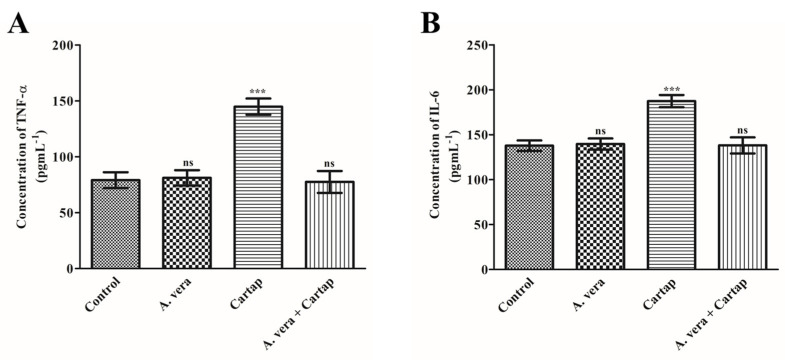
Effects of cartap and *A. vera* on (**A**) TNF-α and (**B**) IL-6, in the serum of rat. The methods for the treatment of cartap and the *A. vera* and the assay for both cytokines were described in Section 2. The unit for the concentration of both cytokines was expressed as pgmL^−1^. The data were significant at *p* ≤ 0.05. Abbreviations: ***: Level of significance; ns: non-significant.

Cytokines are the proteins which are responsible for immunomodulatory effects via the modulation of signaling pathways. The levels of pro-inflamatory cytokines are studied as the markers of inflammatory cellular damage and diseases. The contents of TNF-α and IL-6 were significantly changed in the cartap-treated serum of rats (Figure 3). The concentration of these cytokines was significantly enhanced by 82.92% for TNF-α and 35.99% for IL-6 against cartap. The contents of both pro-inflamatory markers were found to be protected from cartap-mediated toxicity by the *A. vera* extract.

### 3.5. Activities of Transaminases and Phosphatases against Cartap and A. vera

**Figure 4:**
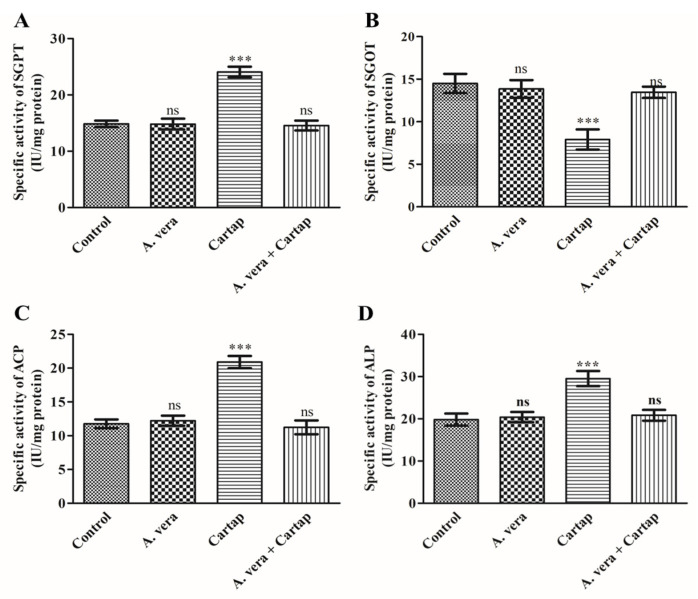
Impact of cartap and *A. vera* on the activities of (**A**): SGPT; (**B**): SGOT; (**C**): ACP; and (**D**): ALP in serum. The methods for cartap and *A. vera* administration and the assays for enzymes were the same as described in Section 2. The units for enzyme activities were presented as IUmg^−1^ protein. Abbreviations: ***: Level of significance; ns: non-significant.

The activity levels of transaminases and phosphatases in serum were monitored as markers of hepatic function. The influence of cartap on liver functions was determined via the activity assays of these enzymes (SGPT, SGOT, ACP and ALP) as described in Materials and Methods. The ameliorating effect of *A. vera* was also assessed. The results indicated an extensive rise in the activity levels of SGPT by 61.86% (Figure 4A) and decline in SGOT by 45.71% (Figure 4B). However, the activity levels of ACP and ALP were also found to be significantly raised by 77.72% and 48.99%, respectively, (Figure 4C and Figure 4D, respectively) in the cartap-treated rats. However, the pre-treatment of *A. vera* showed a non-significant change in the level of enzymes (Figure 4).

### 3.6. Impact of Cartap and A. vera upon AChE Activity in Erythrocytes Membrane and Brain of Rats

**Figure 5:**
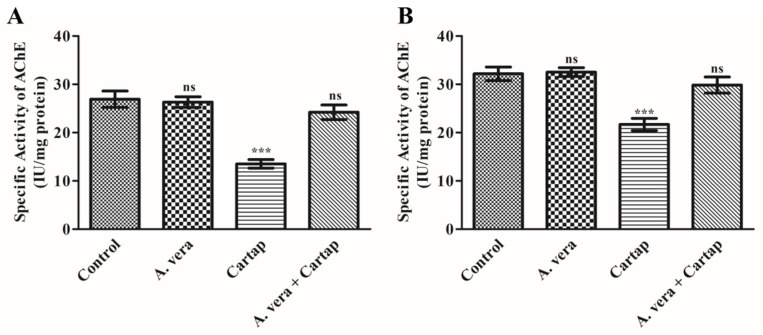
Impact of cartap and the *A. vera* on AChE activity in (**A**): erythrocytes; and (**B**): brain of rats. The methods for cartap and *A. vera* gavage and the activity assay for AChE were as described in Section 2. The unit for AChE activity was presented as IUmg^−1^ protein. Abbreviations: ***: Level of significance; ns: non-significant.

The effects of cartap and *A. vera* were determined by evaluating the activity of AChE, as described in Section 2. The data shown in Figure 5 suggested that cartap treatment substantially (*p* < 0.001) inhibited the AChE activity, doing so by 37.18 % in the erythrocyte membrane (Figure 5A) and 32.62% in the PNS of brain tissue (Figure 5B). This inhibition of AChE was protected by the *A. vera* to near the control level. However, the treatment with *A. vera* only did not cause a substantial change in the activity of AChE.

### 3.7. Impact on the Histology of Rat Liver against Cartap and A. vera

**Figure 6:**
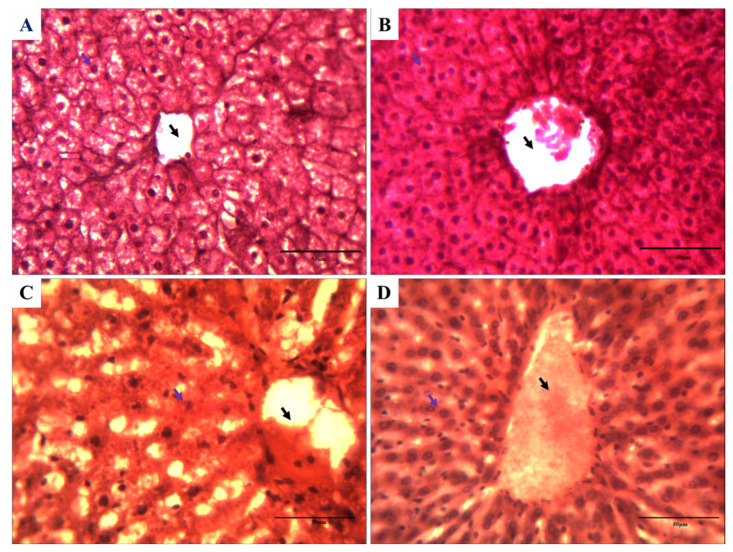
Histological changes in liver due to the exposure cartap and the *A. vera* (H & E staining, X40) to rats. (**A**) Microscpic photograph of liver of control; (**B**) *A. vera* extract-treated; (**C**) cartap-treated (10% LD_50_); and (**D**) *A. vera* + cartap-treated group. The treatment method for cartap and *A. vera* extract were as stated in Section 2. The black arrow indicates the congested central vein, and the blue arrow indicates the disorganized hepatic cord.

After the end of treatment of cartap (10% of LD_50_), the rat liver was excised and processed in order to study the histological aberrations. The aberrations were analyzed using the methods described in Materials and Methods. The light microscopic observations of slides containing liver sections are presented in Figure 6. Figure 6A,B indicate the normal cellular organization of liver tissues. The hexagonal lobules of hepatic tissue were centered on the central vein. It contained a portal triad, having a portal vein, hepatic artery, and bile duct. Figure 6C showed the presence of the disorganization of the central vein and hepatic triad. Liver sections from the *A. vera*-treated group of rats exhibited significant protection (Figure 6D) from the cartap-induced injury to cellular organization. The histological changes in the rat liver were marked and are presented in Table 2.

## 4. Discussion

The massive use of insecticides in fields has led to the exposure of non-target animals to these chemicals, including humans [34], through oral and dermal routes [35]. Pesticide application in farming practice is like a two-edged sword. Pesticides can increase the crop yield by controlling pests; however, it may also pollute water, soil, air, and food grains. The careless use of insecticides has caused several toxicological consequences on the environmental as well as human health [3]. The exposure of living organisms to pesticide has caused impairment in several functions, including cholinergic, cerebral, and hepatic functions [36]. The alteration in these parameters leads to the development of several disorders related to the functions of the markers and immune system [37]. Therefore, there is an urgent need to detect the presence of pesticide [38] and recover these key markers in order to treat hepatic and neurodegenerative disorders [3,19,34,36]. The liver is known for being the hub of the biotransformation of xenobiotics. The pesticide exposure of living systems has been shown to hinder the liver functions and to cause damage to liver and brain tissue [39]. The application of phytochemicals extracted from plants as a therapeutic companion has been shown to contain great promise in this context [40,41,42,43,44].

The exposure to environmental pollutants has been stated to cause oxidative stress and decrease the production of antioxidative agents. Cartap has been reported to cause oxidative stress in several aquatic and terrestrial organisms [15,16]. The establishment of oxidative stress leads to alteration in the ratio of P/A molecules in the cells. The exposure of animals to cartap induces the production of reactive oxygen species, resulting in a significant decrease in the levels of GSH in cells. The oxidation of lipid produces several thiobarbituric acid-reactive substance (TBARS)-like substances, including MDA, that are monitored as the potential markers of oxidative damage [45]. MDA and GSH have been considered potent markers for the analysis of oxidative stress in living organisms [46]. The findings of the present study suggested that the cartap exposure significantly raised the concentrations of MDA and decreased the GSH content in the liver and brain tissue of rats. Our findings corroborated others findings, which have also showed noteworthy rises in the MDA content in rat liver [33] and brain [8] tissue. The present investigation revealed that *A. vera* significantly ameliorated the MDA and GSH levels in hepatic and brain tissue of cartap-exposed rats. The protective nature of *A. vera* extract has been reported to significantly attenuate the altered levels of MDA and GSH in rats [8,33,47,48,49].

The SOD, CAT, and GST enzymes are integral parts of the cellular antioxidant defense system. These enzymes are responsible for converting, quenching, and scavenging of the free radical into non-toxic compounds. These enzymes actively play important roles in maintaining the oxidation–reduction status along with the detoxification of different types of xenobiotics in the biological system. SOD catalyzes the dismutation of s O_2_^•−^ into H_2_O_2_; this can be converted into H_2_O and O_2_ by its reaction with CAT [50]. The GST conjugates the reduced-GSH with xenobiotic compounds, leading to the detoxification of these xenobiotics [51]. Any changes in the activities of these enzymes may cause severe pathophysiological consequences due to the xenobiotic exposure in the key organs of mammals [8,33,49,52]. In the present study, the activities of these enzymes were found to be substantially reduced in the hepatic and brain tissues of cartap-exposed rats. The significant change in the activities of these marker enzymes indicated the collapse of the cellular antioxidant system. Our results are corroborated with studies reported elsewhere on rat treatment with cartap [11,33,49] and other pesticides such as carbofuran [52] and malathion [33,49,53]. However, the *A. vera* extract offered significant amelioration against cartap-induced oxidative stress on the activity levels of SOD, CAT, and GST, as all these enzymes were found to be close to the control.

Proinflammatory cytokines are proteins with low molecular weights. Environmental contaminants have been reported to begin the mass production of cytokines. The level of cytokine production is related to various inflammatory disorders [54]. In the present study, the concentration of TNF-α and IL-6 was found to be markedly enhanced in the cartap-treated group as compared to the control. The xenobiotic-induced stress has been reported to trigger inflammatory reactions by activating different transcription factors [55]. These transcription factors can be activated by enhanced cartap-induced oxidative stress, leading to increased contents of TNF-α and IL-6. The inflammatory response occurs when cells/tissues are injured. These injured tissues/cells may release many chemicals such as bradykinin, prostaglandins, and histamine, thereby producing bulging. This helps researchers to distinguish the inflammation area from the other additional associated body tissues and enhances the activity of phagocytes. Furthermore, the current study also showed the ability of *A. vera* to ameliorate cartap-induced inflammation, as the gavage of *A. vera* substantially protected the levels of both cytokines. The glycoprotein from *A. vera* has been demonstrated to impede the COX-2-mediated inflammatory pathway and reduction in prostaglandin-E2 [56]. The phagocytosis responses may have arisen due to the COX pathway. The *A. vera* has also been revealed to be useful in treating several other inflammatory processes such as burn injury and corneal inflammation [57,58].

The functions of transaminases and phosphatases have been considered as the key markers for the assessment of hepatic cell/tissue damage. Transaminases and phosphatases are known to perform crucial roles in the regulation of physiological processes, such as in the metabolism of xenobiotics and other biomolecules. Whenever the hepatic cells are damaged, the enzymes present in cytosol containing AST, ALT, ACP, and ALP might leak out into the blood. In the present study, the increased activity levels of these enzymes were found in serum of rats treated with cartap, indicating severe injury to hepatocytes. These findings were supported by the histological studies of rat liver tissue. The results from our study were in corroboration with the previous reports [59,60]. The findings from our study demonstrated that *A. vera* extract can substantially protect these enzymes (transaminases and phosphatases) from the cartap-induced adverse effects. The changes in the activity levels of transaminase and phosphatases have been shown to be restored by *A. vera* in cartap-exposed experimental animals [49].

Carbamates has been known to inhibit the cholinergic system because of their capability to specifically inhibit the AChE enzyme [61]. The hydrolysis reaction of acetylcholine catalyzed by the AChE enzyme is important for the normal transmission of nerve signals at the neuronal synapse. The exposure of cartap to the occupationally engaged humans made it necessary to study its impact on AChE activity in the RBC and PNS of brains. The results from current study showed a substantial decline in the levels of AChE extracted from RBC and brain of rats exposed to cartap. The sharp decline in the AChE activity in cartap-exposed rats has indicated its great inhibitory potential. However, the *A. vera* potentially protected the AChE activity against cartap-induced neurotoxicity, as the enzyme function appeared near to the control in RBC and brain of rat. Similar observations were presented by several other researchers when the rats were subjected to cartap exposure and exposed to other carbamates such as carbofuran [8,33,61].

Pesticides are known to induce histological aberrations in liver tissues [33,49,62]. The cartap-exposed rats develop marked damage to the normal cellular architecture of liver tissues, as observed in present study. Our results were supported by the observations recorded by several other investigators [33,49]. The histological analysis displayed that the cartap may cause degeneration of hepatocytes, the hepatic triad, and the central vacuole. In present study, the *A. vera* extract was found to offer protection from the cartap-induced cellular damage to the levels of different histological parameters. These results were supported by earlier reports published by other scholars [8,33].

## 5. Conclusions

The liver and brain comprise the main target organs for chemical if exposed to any xenobiotics, such as pesticides. The liver plays an important function in the detoxification of xenobiotics compounds. Therefore, the liver is the major target organ for various anthropogenic chemicals such as insecticides. The brain has also been proved to be the target of attack if exposed to neurotoxic compounds. The insecticides were reported to drastically disturb the redox status of animals. The findings from the present investigation indicated that the sublethal dose of cartap caused noteworthy changes in oxidative markers, pro-inflamatory markers, and activities of marker enzymes of liver function in Wistar rats. The alterations of these biochemical indices indicated severe damage to hepatic cell/tissue, leading to the altered liver functions. The damage in the tissue was validated by the histopathological studies of liver tissue from Wistar rats. The histology of cartap-treated rat liver revealed severe injury to hepatocytes. The AChE activity was also found to be changed significantly, indicating a decline in the cholinergic functions due to the cartap in rat brain and erythrocyte membrane. The lethal effects of cartap were ameliorated by the *A. vera* extract. The ameliorating properties of *A. vera* extract might be due to the presence of antioxidative molecules in it that may scavenge the free radicals produced by cartap. These results recommended that the ingredients extracted from *A. vera* be utilized as a potential supplement for developing antidotes against pesticides toxicity. The results from this investigation into *A. vera* extract may be exploited in developing safe and cost-effective antioxidants to be used as therapeutic supplements to treat various diseases, including pesticide-induced cerebral and hepatic toxicity, and to develop a better understanding of herbal principles with value-added biomedical activities.

## Figures and Tables

**Table 1:** The levels of OSI (P/A) in the liver and brain under the cartap and *A. vera* exposure in liver and brain tissue.

Parameter	Control	*A. vera*	Cartap	*A. vera* + Cartap
(P/A)_Liver_	0.146	0.147 (+0.68%)	0.434 (+197.26%)	0.152 (+4.109%)
(P/A)_Brain_	0.0560	0.0596 (+6.42%)	0.1285 (+129.46%)	0.0580 (+3.57%)

The OSI was computed as explained in Section 2. The (+) sign showed rise in the level of P/A. Abbreviations: P/A: ratio of prooxidant/antioxidant.

**Table 2:** Effects of cartap and *A. vera* on the cellular architecture of liver tissue sections.

Parameters	Control	*A. vera*	Cartap	*A. vera* + Cartap
DH	_	_	+++	_
DHT	_	_	++++	+
NH	_	_	+++	_
CCV	_	_	+++	_

**Abbreviations:** DH: degenerating hepatocytes; NH: necrosis of hepatocytes; CCV: congestion of central vein; DHT: degradation in hepatic triad; (_): absent; (+): present.

## Data Availability

Not applicable.
